# Distribution and Climatic Adaptation of Wild Tomato (*Solanum lycopersicum* L.) Populations in Mexico

**DOI:** 10.3390/plants11152007

**Published:** 2022-08-01

**Authors:** Gabriela Ramírez-Ojeda, Juan Enrique Rodríguez-Pérez, Eduardo Rodríguez-Guzmán, Jaime Sahagún-Castellanos, José Luis Chávez-Servia, Iris E. Peralta, Luis Ángel Barrera-Guzmán

**Affiliations:** 1 Campo Experimental Centro Altos de Jalisco, Instituto Nacional de Investigaciones Forestales, Agrícolas y Pecuarias (INIFAP), Tepatitlán de Morelos 47600, Mexico; gabramirezo@gmail.com; 2 Departamento de Fitotecnia, Universidad Autónoma Chapingo (UACh), Chapingo 56230, Mexico; jsahagunc@yahoo.com.mx; 3 Centro Universitario de Ciencias Biológicas y Agropecuarias, Universidad de Guadalajara (UdG), Zapopan 45200, Mexico; edrg@hotmail.com; 4 CIIDIR-Oaxaca, Instituto Politécnico Nacional (IPN), Santa Cruz Xoxocotlán, Oaxaca 71230, Mexico; jchavezs@ipn.mx; 5 Facultad de Ciencias Agrarias, Universidad Nacional del Cuyo (UNCUYO), Mendoza M5502JMA, Argentina; iperalta@fca.uncu.edu.ar; 6 Centro Científico Tecnológico CONICET, Instituto Argentino de Investigaciones de las Zonas Áridas, Mendoza C1425FQB, Argentina; 7 Coordinación de Educación e Investigación, Universidad del Valle de Puebla (UVP), Puebla 72440, Mexico; ptc.investigacion3@uvp.mx

**Keywords:** climatic diversity, wild tomatoes, climatic adaptation, *Solanum lycopersicum*

## Abstract

Tomato (*Solanum lycopersicum* L.) is a vegetable with worldwide importance. Its wild or close related species are reservoirs of genes with potential use for the generation of varieties tolerant or resistant to specific biotic and abiotic factors. The objective was to determine the geographic distribution, ecological descriptors, and patterns of diversity and adaptation of 1296 accessions of native tomato from Mexico. An environmental information system was created with 21 climatic variables with a 1 km^2^ spatial resolution. Using multivariate techniques (Principal Component Analysis, PCA; Cluster Analysis, CA) and Geographic Information Systems (GIS), the most relevant variables for accession distribution were identified, as well as the groups formed according to the environmental similarity among these. PCA determined that with the first three PCs (Principal Components), it is possible to explain 84.1% of the total variation. The most relevant information corresponded to seasonal variables of temperature and precipitation. CA revealed five statistically significant clusters. Ecological descriptors were determined and described by classifying accessions in Physiographic Provinces. Temperate climates were the most frequent among tomato accessions. Finally, the potential distribution was determined with the Maxent model with 10 replicates by cross-validation, identifying areas with a high probability of tomato presence. These results constitute a reliable source of useful information for planning accession sites collection and identifying accessions that are vulnerable or susceptible to conservation programs.

## 1. Introduction

Tomato (*Solanum lycopersicum* L.), a member of the *Solanaceae* family, is a worldwide species present in a wide variety of habitats [1] associated with different climate and soil conditions [2]. Mexico and Peru are considered as the possible centers of origin, diversification, and domestication of this species [3,4].

According to Blanca et al. [5,6], the tomato domestication process involved two transitions, the first in South America, which involved the derivation of the partially domesticated species *S. lycopersicum* var. c*erasiforme* (Dunal) D.M. Spooner, G.J. Anderson & R.K. Jansen (SLC) from the wild species *Solanum pimpinellifolium* L. The second transition occurred in Mesoamerica from SLC, which gave rise to the fully domesticated species *S. lycopersicum* var. *lycopersicum* L. as a species with larger fruits. However, Razifard et al. [7] mentioned in recent reports that the origin of SLC is prior to its domestication, as many typical characteristics of tomatoes grown in South America are similar to those of this species. The scarce subsequent presence of SLC was because the partially domesticated forms spread largely.

Mexico is a center of diversification and the most important area in terms of tomato domestication, wild populations are still very frequent, and it is possible to find them in a tolerated, promoted, and even cultivated form [8]. Wild forms of tomato are generally annual or seasonal, although it can manifest as perennial if there are favorable environmental conditions [9,10]. Without favorable environmental conditions, cultivated tomato rarely persists for generations, requiring minimal agronomic management to survive [1,2]. Due to this condition, the conservation of landraces or cultivated forms has occurred mainly by traditional farmers [2,11]. Perturbed areas or natural environments with some degree of disturbance such as agricultural and livestock areas still prevail in several regions of Mexico where wild tomatoes are found [12].

As well as other crops of great worldwide commercial success, tomato has lost genetic variability during the domestication process [13,14], especially for genetic breeding and the development of new commercial varieties with adaptation and tolerance to adverse abiotic factors [15] and pathogens in pre- and post-harvest [16]. A way to recover this genetic variability loss is through native, wild, and related species germplasms to incorporate specific agronomic and fruit quality traits into commercial varieties [17], through programs for the enhancement of crop wild relatives (CWRs), as has already started in other parts of the world, in order not to lose those fundamental genetic characteristics [18].

In Mexico, there are valuable genetic pools of wild local naturalized tomato populations detected from studies that demonstrate diversity at both morphological and molecular levels [19], as well as variations in the tolerance to nematodes [20]. Likewise, quality characteristics of the fruit have shown great variation, for example, antioxidant capacity and isoprenoids metabolism [21]; concentrations of nutraceuticals and antioxidant compounds such as vitamin C, lycopene, organic acids, and soluble solids [22,23,24]; concentrations of sugars, carotenoids, carotenoid-derived volatiles, and consumer preference flavor and aroma [24]. In addition, the variations in size, shape, color, fruit flavor, postharvest quality, culinary characteristics [23,24], and hedonic quality [25] are high. Despite this, studies remain scarce given the vast diversity of tomato genetic resources in Mexico. Therefore, there is still a need to collect, characterize, conserve, and use them in a sustainable way through in situ and ex situ conservation.

To achieve these purposes, it is essential to identify the status of the current potential distribution of wild tomato populations in Mexico based on diversity and climatic characterization, information that currently does not exist. This information will allow the determination of its spatial and temporal disposition, the history and the dynamics of its development [26,27], and with this, a better understanding of the interactions between environmental conditions and biotic and abiotic factors with which this species has co-evolved [28]. To perform this task, the use of geographic information system (GIS) tools is required, allowing the observation, capture, entry, storage, and analysis of data for decision-making [27].

Due to the above, the objective of this research was to determine the current distribution areas of wild and native tomato populations in Mexico by identifying their adaptive ranges and climatic adaptation patterns, through the application of ecogeographic methods carried out with GIS tools and multivariate analysis.

## 2. Results

### 2.1. Statistical Analyses and Ecological Descriptors

In order to eliminate collinearity between the variables used and avoid erroneous statistical results, the selection of variables was made based on Pearson correlation coefficients, discarding those variables with absolute coefficients ≥ 0.9. The selected variables for subsequent analysis were ET (annual evapotranspiration, mm), ALT (digital elevation model, m), B1 (annual mean temperature, °C), B2 (mean diurnal range, °C), B3 (isothermality (B2/B7) × (100)), B4 (temperature seasonality, standard deviation × 100), B7 (temperature annual range, °C), B12 (annual precipitation, mm), B14 (precipitation of driest month, mm), and B15 (precipitation seasonality, coefficient of variation).

**Figure 1:**
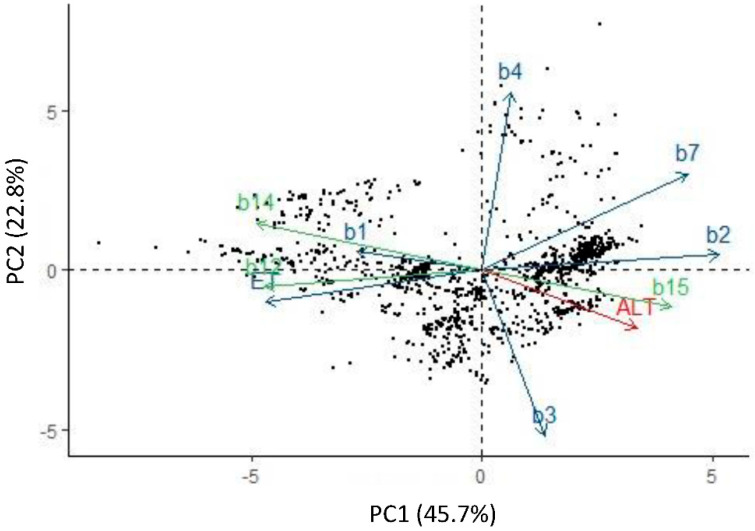
Biplot based on 10 climatic variables and 1296 accessions of *S. lycopersicum* in Mexico (black dots). PC1 and PC2 explained 45.7 and 22.8% of the total variation, respectively. B1 (annual mean temperature), B2 (mean diurnal range), B3 (isothermality), B4 (temperature seasonality), B7 (temperature annual range), B12 (annual precipitation), B14 (precipitation of the driest month), B15 (precipitation seasonality), ET (annual evapotranspiration), and ALT (digital elevation model).

Regarding statistical analyses, Principal Component Analysis (PCA) determined that three principal components (PCs) described 84.1% of the total variation in the data from the accession sites. PC1 captured 45.6% of the total variation and had a greater linear association with B2, B7, B12, B14, and ET. On the other hand, B3 and B4 excelled on PC2 that corresponds to 22.8% of the total variation (Figure 1). Altitude through the digital elevation model and annual mean temperature were represented in PC3, which captured 15.7% of the total data variation.

**Figure 2:**
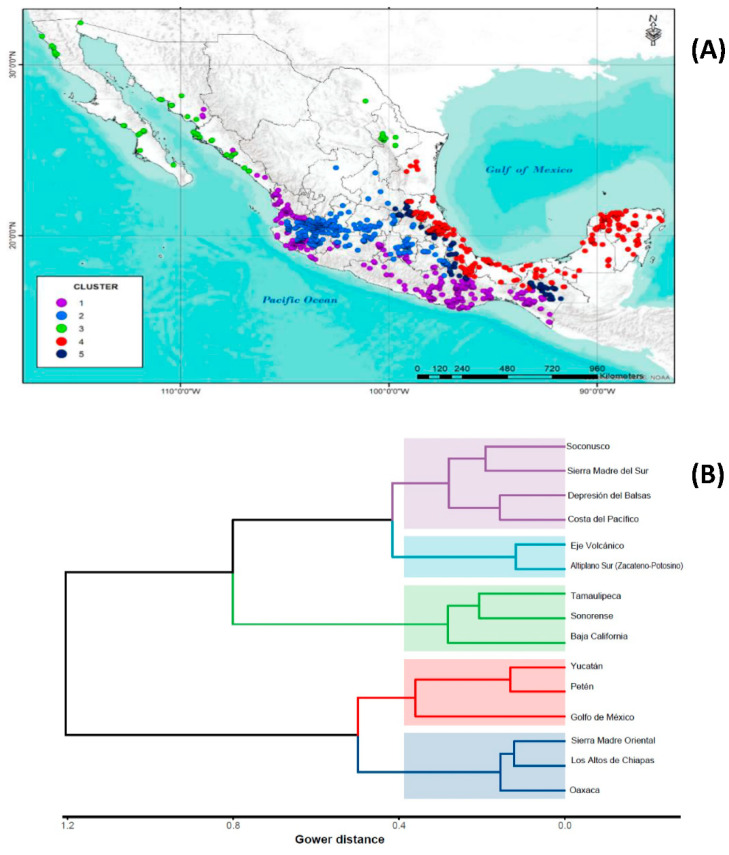
(**A**) Map with the geographical distribution of *S. lycopersicum* L. accessions in Mexico according to 5 clusters identified in CA. (**B**) Dendrogram with Gower distances and Ward’s clustering method.

Cluster analysis (CA) was performed in order to group geographic provinces based on the 10 climatic variables considered and Gower distances. The Hopkins statistic indicated that the clustering trend in the dataset corresponded to a normal distribution and showed evidence (H = 0.073) that there are real clusters. The dendrogram construction algorithm was k-means, chosen according to the result of clvalid. The Nbclust algorithm determined that the number of optimal groups was 5 (Figure 2).

Thus, the wild populations of *S. lycopersicum* are distributed in the following 15 of the 19 physiographic provinces of Mexico : Altiplano sur (Zacatecano-Potosino), Baja California, Costa del Pacífico, Depresión del Balsas, Eje Volcánico, Golfo de México, Los Altos de Chiapas, Oaxaca, Petén, Sierra Madre del Sur, Sierra Madre Oriental, Soconusco, Sonorense, Yucatán, and Tamaulipeca.

Regarding the five identified clusters, they present a well-defined geographical distribution (Figure 2). Cluster 1 is made up of accessions from the Pacific coastal zone from southern Sonora to Chiapas. This group is distinguished by having the largest distribution in the country, as well as the greatest amplitude in the climatic ranges and ecological descriptors of all the variables used in the analysis (Table 1).

Cluster 2 was located along the transvolcanic zone of the country. These accessions are those with the highest altitude range and the lowest annual mean temperature. Cluster 3 contains regions located in the northern part of the country with the lowest annual precipitation and evapotranspiration values. Cluster 4 identifies regions located along the coast of the Gulf of Mexico, from Tamaulipas to Yucatán, with a tendency to present low altitude, high annual mean temperature, annual precipitation, and evapotranspiration. Finally, cluster 5 corresponds to the mountainous area in the east of the country, from the south of San Luis Potosí to Chiapas with environments with high altitude, low annual mean temperatures, and high water availability.

**Figure 3:**
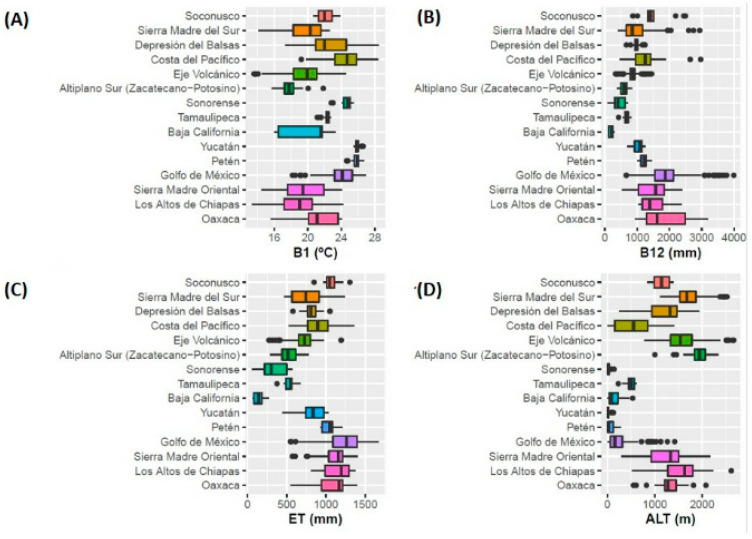
Boxplots of four important variables for the distribution of wild tomato species in Mexico. (**A**) Annual mean temperature (B1, °C), (**B**) annual precipitation (B12, mm), (**C**) annual evapotranspiration (ET, mm), and (**D**) digital elevation model (ALT, m).

Regarding ecological descriptors shown in Table 1 and Figure 3, the climatic amplitude and the well-defined correspondence of the wild tomato collection sites distributed in the different physiographic provinces can be observed and grouped according to the dendrogram generated by CA. Among the main findings, wild tomato populations in the Gulf of Mexico area stand out, where the highest annual precipitation and evapotranspiration values occur. In contrast, the populations of the Baja California area face water scarcity. The accessions of the province of Yucatán face high values of annual mean temperature and lower altitude, while those in the area of Altiplano Sur (Zacatecano-Potosino) withstand lower annual mean temperatures associated with higher altitudes.

### 2.2. Climatic Diversity and Hotspot Analysis

Beck et al. [29] reported 30 climate types using as references the Köppen–Geiger system. Among tomato accessions in Mexico, only 10 of them were identified: (Af, Am, Aw, BWh, BSh, BSk, Cwa, Cwb, Cfa, and Cfb), with a predominance of temperate climates (C). BSh (arid, steppe, hot) and Aw (tropical, savannah) climates were the ones present in most of the previously identified physiographic provinces. Af climate type (tropical, rainforest) was the one with the least abundance, appearing only in the accessions of the region of Gulf of Mexico and Oaxaca.

**Figure 4:**
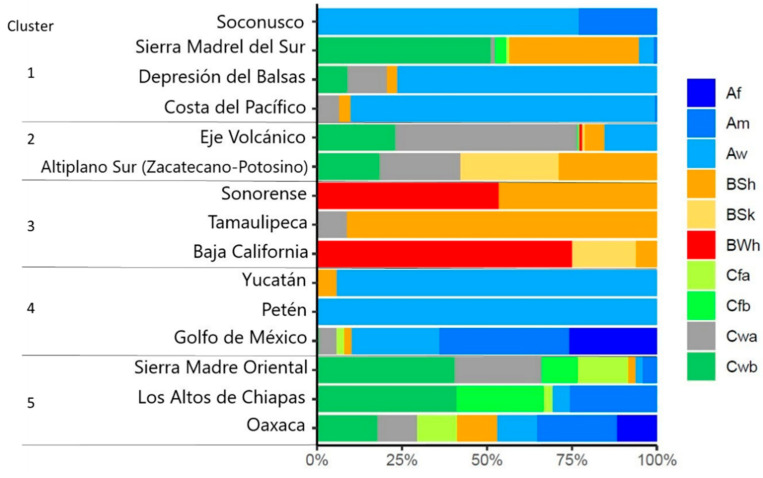
Climatic diversity and climate type percentages for tomato accessions in Mexico by physiographic province according to the groups formed by CA. Climate types according to Beck et al. [29]. Af (Tropical, rainforest), Am (Tropical, monsoon), Aw (Tropical, Savannah), BWh (Arid, desert, hot), BSh (Arid, steppe, hot), BSk (Arid, steppe, cold), Cwa (Temperate, dry winter, and hot summer), Cwb (Temperate, dry winter, and warm summer), Cfa (Temperate, no dry season, and hot summer), and Cfb (Temperate, not dry season, warm summer).

Regarding climate diversity among accessions located in each physiographic province, accessions of the Petén region were those that were in a single climatic type (Aw, tropical, Savannah). Accessions from Sierra Madre del Sur region, the Eje Volcánico, Gulf of Mexico, Sierra Madre Oriental, and Oaxaca are distributed in the greatest diversity of climates (seven climatic types). Figure 4 shows the distribution of climatic diversity for each province according to the clusters previously identified in CA. In this graph, it is possible to observe that, within each group, there is considerable climatic similarity with a different proportion between the physiographic provinces that make up each cluster.

**Figure 5:**
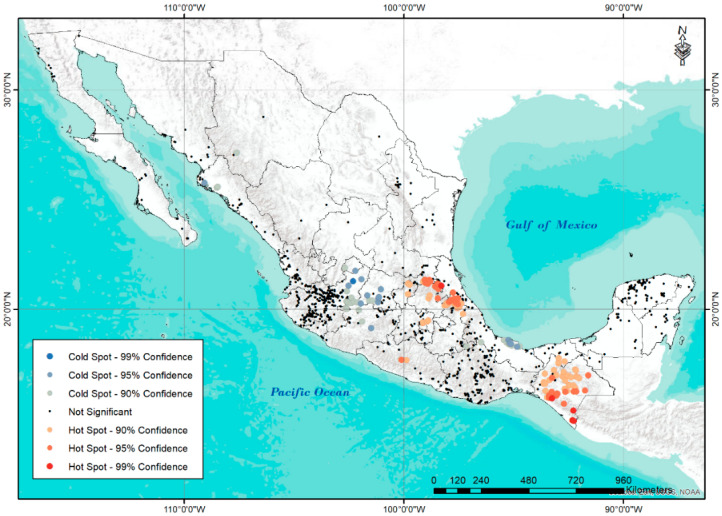
Abundance map of hotspot (red) and coldspot (blue) for 1296 *S. lycopersicum* L. accessions in Mexico.

On the other hand, hotspot analysis (Figure 5) revealed the presence of zones of high and low diversity in a satisfactory manner. Among the areas with the greatest diversity or hot zones are practically all the accessions of Chiapas; the border area between Veracruz with Hidalgo, Querétaro, and Puebla; finally, a small area of the State of Mexico adjoining CDMX, as well as another small area of the state of Guerrero. Regarding the areas of importance due to the low diversity of accessions or coldspots, the northern zone of Sinaloa, the south of Veracruz, and the northern zone of Jalisco are located, bordering Guanajuato and Michoacán.

### 2.3. Potential Distribution

**Figure 6:**
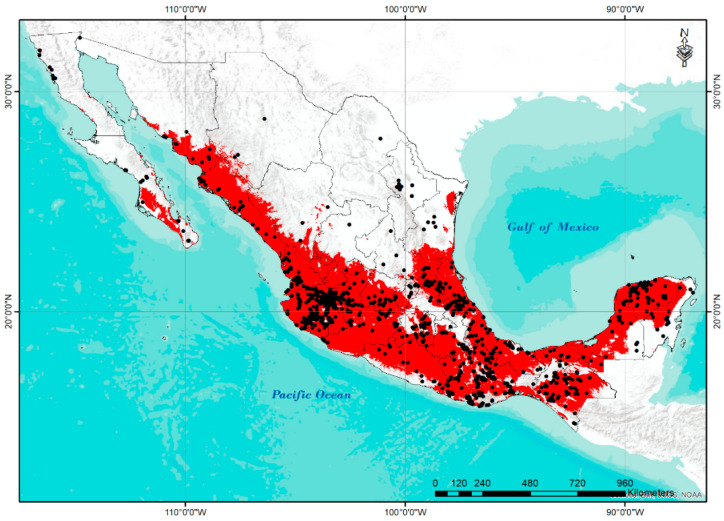
Potential distribution of *S. lycopersicum* in Mexico. Red represents potential distribution area; black dots represent all *S. lycopersicum* accessions.

Figure 6 represents the potential distribution of *S. lycopersicum* L. in Mexico obtained using the Maxent V 3.4.4 model [30]. The map is an ensemble model of the average value of 10 replicates by cross-validation. The threshold value chosen according to the rule of low omission rate over the maximum logistic value in all replicates was 10 percentile training presence, with an average value of 0.358.

The potential distribution model of tomato had an adequate fit and performance, shown by the average value of AUC (0.932), which allows it to strongly discriminate the suitable areas from those not suitable for the distribution of wild populations of this tomato species [31]. Additionally, this model shows a very close coincidence with the regions where the collection sites are located, with the exception of the northern part of the country (Baja California, and some accessions in Chihuahua, Coahuila, and Tamaulipas).

## 3. Discussion

Mexico, as a center of tomato domestication, presents great variability in its wild populations growing as naturalized and ruderal plant [8,32]. This is due to the varied orography and climate conditions represented by the 19 physiographic provinces of this country. Thus, given the great variability of environmental conditions that wild populations faced during the processes of natural selection, they necessarily developed adaptations to adverse conditions, which makes them a valuable genetic resource for direct use or in the generation of new improved varieties. Even with the great climate diversity in Mexico, only one tomato species with two varieties is found naturally (*S. lycopersicum* var. *cerasiforme* and *S. lycopersicum* var *lycopersicum*).

Despite this recognized importance, information about the current geographical distribution of wild tomato in Mexico and the ecological requirements that determine its distribution is partial or incomplete. This information is relevant to formulate effective strategies for sustainable use and conservation of plant genetic resources, which must be in line with the specific characteristics of the species, of the habitats in which they grow, and with world laws on the conservation of genetic resources [33].

One threat to this vast tomato genetic diversity is climate change. For the 21st century, it is predicted that there will be significant modifications in the current thermal and rainfall patterns, causing extreme variations that will severely affect natural systems [34,35]. These weather modifications will strongly alter the geographic distribution of the species, positioning plant genetic resources as a highly vulnerable sector to the impacts produced by this phenomenon [35,36].

Regarding the results of this research, the identification of multicollinear variables through Pearson correlation allowed the elimination of 11 of the 21 variables initially considered, implying that the variation detected in them can be described by the chosen variables. This is because some variables are indices that share basic information to obtain them, hence the high values of correlations, which implies linear-type associations. Therefore, by eliminating “artificial variation”, the performance of multivariate analyses (CA and PCA) was improved [37].

PCA made it possible to describe 84.1% of the data variation with three PCs. Unlike the results obtained by Ramirez et al. [38] on the climatic variables of greatest importance for the diversity and distribution of wild and related tomato species in Latin America, the variables that make up each PC are not grouped uniformly, i.e., in each component, there are variables related to temperature and humidity. Likewise, variables such as altitude and annual mean temperature, considered very relevant for the distribution of tomato species [38,39], were integrated in the third component. Given these results, it can be assumed that the distribution of wild tomato in Mexico is determined by the presence of thermal and pluvial factors in the same proportion, unlike the 12 wild and four related species distributed in Latin America that are more affected by precipitation [38,39]. These changes in the distribution associated with climatic variables can be attributed to the tomato domestication process.

Groups formed by CA based on the criterion of distribution in physiographic provinces (Figure 2B) made it possible to identify populations of tomato accessions adapted to diverse environmental conditions, locating zones with the presence of germplasms tolerant to specific factors. Information generated about ecological descriptors associated with physiographic provinces (Table 1) is a source of information of great importance, as combining them opens the possibility of searching for specific tolerance or resistance genes to adverse environmental factors (extreme temperatures, drought, excess of humidity, salinity, and presence of diseases), which is very useful for genetic breeding of tomato commercial varieties. This information, together with all the agronomic information generated over the years, is of great help for the identification and selection of materials with potential use for genetic breeding programs [40,41,42,43].

Regarding climate diversity, the predominant climate type in the wild populations of Mexico is temperate (C), unlike the Latin American tomato species where dry climates predominate (B) [39]. This condition is also favorable to know the environmental suitability of each group of species. 

Hotspot analysis (Figure 5) results satisfactorily showed the areas of high diversity of wild tomato populations into areas with high-diversity climate conditions. These zones coincide with known areas of great diversity and the use of wild tomatoes [8]. Low-diversity zones or coldspot areas are important sites to consider for the conservation of these resources.

The Maxent model used to determine the potential distribution of tomato in Mexico has been recognized for its efficiency in handling complex interactions between predictor and response variables [28,44,45]. The coincidence between the presence of wild tomato populations and those predicted by the distribution model are very close in areas where the crop is widely adapted. As environmental conditions become more stressful for wild populations, the model loses sensitivity and efficiency as environmental conditions and, consequently, the response of the populations are much more variable and strongly limit the prediction of their distribution. However, in areas where environmental conditions correspond to the needs of wild populations, the predictive performance of this distribution model is commendable. The lack of coincidence in some regions of the north of Mexico between accessions and the predicted distribution area is assumed to be due to the date of collection of accessions (accessions collected a long time ago and that generates uncertainty about their current presence), and that the current climatic conditions are no longer the most favorable for the development of the species.

The present study constitutes a reliable source of information for the generation of strategies for sustainable use and conservation of tomato genetic resources in Mexico. However, it is still necessary to evaluate the impact of climate change on the distribution of these populations, effects on genetic diversity, and agricultural systems [46]; together with the information generated in this research, it will be possible to design future collection routes for conservation and use of these resources.

## 4. Materials and Methods

### 4.1. Database

**Figure 7:**
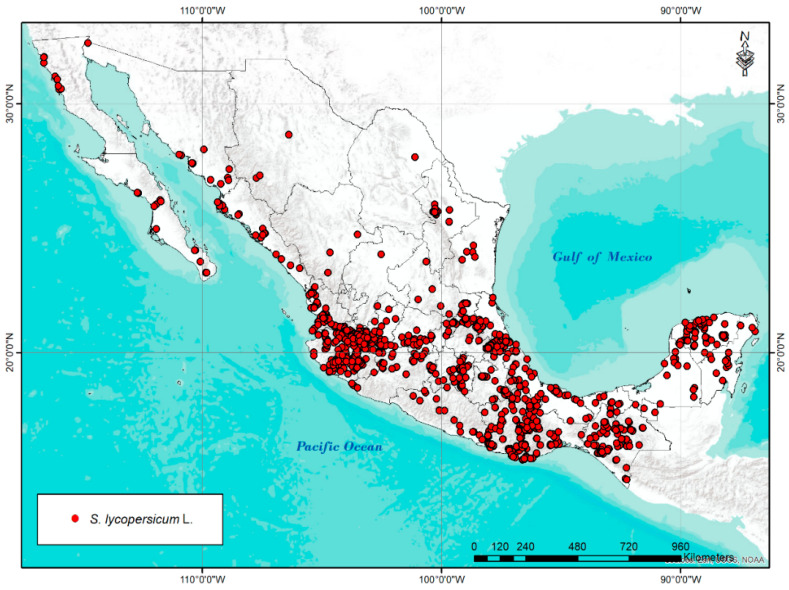
Geographical distribution of 1296 collections of *S. lycopersicum* L. in Mexico.

The database with which this research was carried out was integrated with geo-referenced passport data of *S. lycopersicum* L. in Mexico. For this purpose, 2983 geographic coordinates were identified from: the National Network of Tomato-SNICS, germplasm banks (National Center of Genetic Resources-INIFAP, National Bank of Plant Germplasm-UACH), herbarium specimens (Herbarium of the University of Guadalajara, Herbarium of the University of Science and Arts of Chiapas, Herbarium of the Colegio de la Frontera Sur, and Herbarium of the Institute of History and Ecology of Chiapas), reports and scientific articles [40,41,42,43], and national (Global Biodiversity Information Network) [47] and international plant inventories (Tomato Genetics Resource Center and Global Biodiversity Information Facility) [48,49]. Of the total number of accessions collected, records with atypical data, repeated records, records with geographic coordinates with low precision (less than 3 decimal places), and accessions located in atypical areas were discarded, leaving a total of 1296 records (Figure 7). It is necessary to mention that the collections were identified at the level of taxonomic variety due to little or no information on some of the records used.

### 4.2. Environmental Information

A climate information system with 20 climatic and one geographic variables was used in raster format with a spatial resolution of 1 km^2^. Bioclimatic variables belong to WorldClim version 2.1 corresponding to the period of 1970–2000 [50]: B1 (Annual mean temperature, °C), B2 (Mean diurnal range, °C), B3 (Isothermality), B4 (Temperature seasonality), B5 (Maximum temperature of warmest month, °C), B6 (Minimum temperature of coldest month, °C), B7 (Temperature annual range, °C), B8 (Mean temperature of wettest quarter, °C), B9 (Mean temperature of driest quarter, °C), B10 (Mean temperature of warmest quarter, °C), B11 (Mean temperature of coldest quarter, °C), B12 (Annual precipitation, mm), B13 (Precipitation of wettest month, mm), B14 (Precipitation of driest month, mm), B15 (Precipitation seasonality), B16 (Precipitation of the wettest quarter, mm), B17 (Precipitation of the driest quarter, mm), B18 (Precipitation of the warmest quarter, mm), and B19 (Precipitation of the coldest quarter, mm). Additionally, annual evapotranspiration (ET, mm) was obtained from the sum of the monthly values, according to Trabucco and Zomer [51]. The geographic variable altitude was determined with a digital elevation model for every accession site [50].

**Figure 8:**
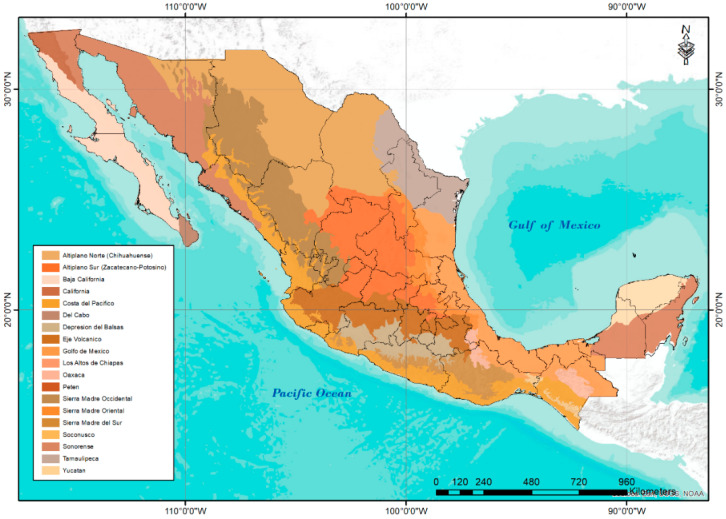
Physiographic Provinces of Mexico proposed in 2016 by “Comisión Nacional para el Conocimiento y Uso de la Biodiversidad” (CONABIO) [52].

In order to make information more understandable, accession sites were grouped according to the classification of physiographic provinces of Mexico [52] (Figure 8). This information was used to identify climatic patterns and diversity, perform statistical analysis, and define ecological descriptors.

### 4.3. Statistical Analysis and Ecological Descriptors

Before running statistical analyses, a selection of variables was made in order to identify high linear dependence (collinearity) among more than two variables. This selection of variables was obtained with Pearson correlations between variables, eliminating one of those two variables with absolute coefficients greater than 0.90.

Principal Component Analysis (PCA) with a correlation matrix was performed with the selected variables in SAS V 9.4 (PRINCOMP procedure) [53]. All graphics were elaborated in RStudio [54] (Figure 2B, Figure 3 and Figure 4). Eigenvalues, eigenvectors, and the contribution of the variables for each principal component for the corresponding figures were obtained with the packages FactoMineR [55] and Factoextra [56].

Subsequently, Cluster Analysis (CA) with Euclidean distances and Ward’s lower variance clustering method was run to identify similar accessions by physiographic provinces. The clustering tendency was verified with Hopkins (H) statistic with the clustertend package [57] where values greater than or equal to 0.5 indicate that they are very close and the data are uniformly distributed, so clustering does not make sense; values close to 0 are evidence in favor of clustering of the data. The best algorithm for clustering was calculated with the clValid package [58]. The selection of the optimal number of clusters was determined with the NbClust package [59].

Ecological descriptors were calculated using the methodology proposed by Steiner and Greene [60], and widely used in different species [28,38,39,61,62].

To determine ecological descriptors, a vector of points with the geographic coordinates of each accession was used and the values of each variable were determined. These values were obtained with the Spatial Analyst Tools of ArcGIS (software GIS) version 10.3, ESRI Inc., Redlands, CA [63]. The information was concentrated in an Excel spreadsheet where the extreme values or range (minimum and maximum), median, and coefficient of variation (CV = [Q/Med] × 100, where Q = [Q3 − Q1]/2 (interquartile range), and Med = median) of each accession were determined [38]. This process was carried out for each of the selected variables.

### 4.4. Climate Diversity and Hotspot Analysis

Climate diversity patterns were identified by taking into account physiographic provinces in Mexico by “Comisión Nacional para el Conocimiento y Uso de la Biodiversidad” (CONABIO) [52]. For this analysis, the geographical coordinate vectors of each accession were used and the climate type was obtained (Table 2) according to the world climatic classification with the Köppen–Geiger system with a spatial resolution of ~1 km^2^ proposed by Beck et al. [29]. With the information obtained, a frequency table was integrated identifying the number of accessions for each climate type in each physiographic province identified in the accession areas.

For hotspot analysis, critical zones of species abundance and areas with a high concentration of diversity were identified using the “Spatial Statistics Tools” of ArcGis.

Species density maps were constructed by identifying all accessions within 1 km of each other. This distance was chosen based on previous studies of diversity in wild tomato species in South America [39] and potato species (Solanum Sect. Petota), a sister group of tomatoes [64,65].

Hotspot analysis was performed to determine the hot or cold spatial clustering of collections as expected with a random distribution. The analysis was run with the Getis-Ord Gi* statistic [66] to quantify specific regions of high clustering and spatial significance for accessions abundance and diversity. Statistical significance of the analysis was calculated using Z values.

### 4.5. Potential Distribution

The potential distribution model for tomato in Mexico was determined with MaxEnt model V. 3.4.4 [30], which is based on the principle of maximum entropy to estimate a set of functions that relate the suitability of the environment to environmental variables and determine the potential distribution of a species [31]. The Maxent model has been recognized for its efficiency in handling complex interactions between predictor variables and response variables [28,44,45].

Regarding the model parameters, the occurrence data were randomly divided into training data (50%) and test data (50%) in order to test the fit and statistical significance of the model [67]. Finally, the model output was presented as the ensemble model of 10 replicates by cross-validation.

The model performance was evaluated by estimating the area under the curve (AUC) from plots of receiver operating characteristics [68]. Such a statistic is useful to evaluate the goodness of selection of suitable versus unsuitable areas for tomato distribution, where models with an AUC greater than 0.7 are acceptable and perform well [28,69].

The resulting ensemble model was presented as a binomial presence/absence map for tomato distribution by choosing the threshold value of environmental fitness by selecting the threshold value (Fixed cumulative value 1, Minimum training presence, 10 percentile training presence, and maximum training sensitivity plus specificity) that guarantees the lowest omission rate (known areas of predicted occurrence/absence) at a maximum logistic value [28].

## Figures and Tables

**Table 1:** Ecological descriptors of 10 climatic variables for *S. lycopersicum* L. distribution in Mexico according to physiographic provinces and cluster groups identified in CA. Range (Minimum–Maximum value), Med (Median), CV (Coefficient of variation), B1 (annual mean temperature), B2 (mean diurnal range), B3 (isothermality), B4 (temperature seasonality), B7 (temperature annual range), B12 (annual precipitation), B14 (precipitation of the driest month), B15 (precipitation seasonality), ET (annual evapotranspiration), and ALT (digital elevation model).

CLUSTER	PHYSIOGRAPHIC PROVINCE	PC1														
		B2			B7			B12			B14			B15		
		Range	Med	CV	Range	Med	CV	Range	Med	CV	Range	Med	CV	Range	Med	CV
**1**	Soconusco	11.5–13.3	12.3	3.14	16.0–20.1	17.1	3.22	865–2497	1407	6.04	0–17	2	250	65.5–108.8	105.3	5.2
	Sierra Madre del Sur	9.4–17.8	13.6	14.8	14.4–24.6	19.6	13	414–2938	849	29.93	01–64.0	5	65	76.1–105.3	92.5	2.4
	Depresión del Balsas	13.8–17.3	15.4	4.41	19.8–25.9	23.2	4.71	665–1240	969	3.99	1.0–9.0	3	33.33	93.8–119.5	108.4	1.07
	Costa del Pacífico	9.6–19.1	13.8	10.66	12.8–31.9	20.1	14.17	466–2968	1248	18.96	0–15.0	2	50	82.4–133.9	112.7	5.81
**2**	Eje Volcánico	11.9–19.5	16.9	5.19	18.6–31.0	26.1	5.94	327–1449	844	8.12	0–36.0	4	37.5	73.6–124.7	110.4	5.87
	Altiplano Sur	13.4–18.7	16.5	6.12	21.2–29.4	25.8	8.43	367–835	592	15.41	2.0–11.0	5	20	67.8–118.3	94.4	15.08
**3**	Sonorense	12.0–17.9	14.8	4.16	23.7–35.4	25.7	6.6	78–722	402	39.86	0–2.0	1	37.5	49.7–122.4	115.2	4.4
	Tamaulipeca	13.5–15.1	14.3	0.76	27.1–31.8	27.8	2.43	424–815	696	7.51	11.0–21.0	18	5.56	58.6–85.0	80.7	5.47
	Baja California	11.6–18.5	15.9	8.74	19.3–28.6	26.8	9.51	102–294	205	31.52	0–1.0	0	0	70.5–124.8	89.9	7.53
**4**	Yucatán	10.7–14.9	13.4	4.34	16.1–22.0	19.8	3.47	694–1253	1075	10.57	16.0–39.0	23	8.15	59.1–87.6	67.6	4.78
	Petén	9.7–14.5	12.4	6.49	14.5–20.6	17.5	7.43	1005–1449	1230	6.12	18–43	37	19.57	47.6–84.5	56.1	5.4
	Golfo de México	7.9–14.1	10.5	6.98	15.2–27.7	18.3	5.74	657–3995	1867	15.63	8–141	49.5	26.01	42.4–111.8	66	15.76
**5**	Sierra Madre Oriental	9.8–14.6	12	3.92	17.3–25.2	20.5	5.37	525–2404	1573	24.95	8.0–56.0	37	25.68	67.4–90.8	78.9	5.59
	Los Altos de Chiapas	10.7–14.1	12.5	6.07	16.8–19.7	18.7	2.81	1045–2376	1390	22.72	3.0–73.0	22	55.68	55.0–93.7	82.4	9.82
	Oaxaca	11.0–16.7	13.1	9.5	17.6–24.8	20.5	7.32	923–3192	1616	36.88	11.0–72.0	34	57.35	70.1–92.4	79.3	9.12
**CLUSTER**	**PHYSIOGRAPHIC PROVINCE**	**PC1**			**PC2**						**PC3**					
		**ET**			**B3**			**B4**			**B1**			**ALT**		
		**Range**	**Med**	**CV**	**Range**	**Med**	**CV**	**Range**	**Med**	**CV**	**Range**	**Med**	**CV**	**Range**	**Med**	**CV**
**1**	Soconusco	3–121	13	134.6	66.3–75.3	72.1	2.38	93.6–173.8	134	6.63	20.7–23.9	21.9	3.62	839–1396	1141	14.02
	Sierra Madre del Sur	12–310	26	52.4	61.2–79.4	71.5	2.98	83.9–197.8	143	12.6	14.2–22.6	20.3	8.09	1108–2523	1676	10.02
	Depresión del Balsas	18–21	29	12.07	64.4–69.9	67	1.76	135.9–213.8	183	5.01	17.3–28.4	21.9	8.28	243–1940	1320	19.95
	Costa del Pacífico	1–99	37	52.7	55.1–80.0	68.5	3.22	61.4–486.2	175	34.08	19.2–28.4	24.6	5.24	5–1406	550	63.51
**2**	Eje Volcánico	9–122	34	20.59	59.8–73.0	64.4	2.57	124.3–309.0	240	9.62	13.6–24.5	19.9	6.99	773–2667	1539	14.62
	Altiplano Sur	21–46	27	10.65	59.6–70.1	63.5	1.75	173.6–349.7	272	8.32	15.7–21.9	17.7	3	1002–2344	1939	5.75
**3**	Sonorense	28–64	52	6.73	46.6–61.9	55.2	6.37	368.6–742.4	487	14.79	22.8–25.5	24.7	1.49	1–139	15	86.67
	Tamaulipeca	43–73	60	4.17	46.3–53.2	50.6	1.44	485.3–644.1	504	3.72	21.1–22.7	22.3	0.76	229–613	504	12.05
	Baja California	32–155	54	63.43	56.8–65.9	60.8	2.72	279.2–499.4	368	13.44	16.0–23.3	21.5	12.17	4–532	86	108.2
**4**	Yucatán	78–150	100	7.71	63.9–71.5	67.5	1.58	181.8–218.1	196	2.85	25.4–26.6	25.8	0.52	0–124	15	64.6
	Petén	89–203	147	10.63	63.8–74.1	70.3	1.91	154.3–215.4	185	7.01	24.6–26.7	25.9	0.96	6–279	22	243.2
	Golfo de México	31–836	209	23.33	49.5–68.0	56.1	4.47	176.9–509.0	286	16.05	18.2–26.9	24.1	4.34	0–1419	156	79.63
**5**	Sierra Madre Oriental	34–185	122	24.18	53.2–63.0	59.4	3.21	183.3–368.4	237	20.54	14.5–24.1	19.4	11.09	299–2166	1330	21.71
	Los Altos de Chiapas	20–328	87	67.82	62.5–71.9	67.3	3.84	133.6–203.7	169	7.79	13.4–24.2	19.1	8.83	518–2613	1629	16.04
	Oaxaca	45–290	128	60.16	60.3–68.0	64.8	3.26	191.0–238.9	219	2.82	15.6–24.1	21.1	8.38	549–2078	1275	9.33

**Table 2:** Climate types according to Beck et al. [29] to determine diversity patterns among wild tomato accessions *S. lycopersicum* L. in Mexico.

Climate Type
**Af** (tropical and rainforest), **Am** (tropical and monsoon), **Aw** (tropical and savannah), **BWh** (arid, desert, and hot), **BWk** (arid, desert, and cold), **BSh** (arid, steppe, and hot), **BSk** (arid, steppe, and cold), **Csa** (temperate, dry summer, and hot summer), **Csb** (temperate, dry summer, and warm summer), **Csc** (temperate and dry and cold summer), **Cwa** (temperate, dry winter, and hot summer), **Cwb** (temperate, dry winter, and warm summer), **Cwc** (temperate, dry winter, and cold summer), **Cfa** (temperate, no dry season, and hot summer), **Cfb** (temperate, no dry season, and warm summer), **Cfc** (temperate, no dry season, and cold summer), **Dsa** (cold, dry summer, and hot summer), **Dsb** (cold, dry summer, and warm summer), **Dsc** (cold, dry summer, and cold summer), **Dsd** (cold, dry summer, and very cold winter), **Dwa** (cold, dry winter, and hot summer), **Dwb** (cold, dry winter, and warm summer), **Dwc** (cold, dry winter, and cold summer), **Dwd** (cold, dry winter, and very cold winter), **Dfa** (cold, no dry season, and hot summer), **Dfb** (cold, no dry season, and warm summer), **Dfc** (cold, no dry season, and cold summer), **Dfd** (cold, no dry season, and very cold winter), **ET** (polar and tundra), and **EF** (polar and frost).

## Data Availability

All data sources supporting accessions and climatic information used are mentioned in the Methodology section: GBIF (https://www.gbif.org, accessed on 25 January 2022); TGRC (https://tgrc.ucdavis.edu, accessed on 27 January 2022); WorldClim (https://www.worldclim.org/, accessed on 25 January 2022).
